# Efficacy and effectiveness of infant vaccination against chronic hepatitis B in the Gambia Hepatitis Intervention Study (1986–90) and in the nationwide immunisation program

**DOI:** 10.1186/1471-2334-14-7

**Published:** 2014-01-07

**Authors:** Thomas J Peto, Maimuma E Mendy, Yamundow Lowe, Emily L Webb, Hilton C Whittle, Andrew J Hall

**Affiliations:** 1 Medical Research Council Laboratories, Fajara, The Gambia; 2 London School of Hygiene and Tropical Medicine, London, UK; 3 Mahidol-Oxford Tropical Medicine Research Unit, Faculty of Tropical Medicine, Mahidol University, 420/6 Rajvithi Rd., Bangkok, 10400, Thailand; 4 International Agency for Research on Cancer, Lyon, France; 5 Ministry of Health and Social Welfare, Banjul, The Gambia

## Abstract

**Background:**

Gambian infants were not routinely vaccinated against hepatitis B virus (HBV) before 1986. During 1986–90 the Gambia Hepatitis Intervention Study (GHIS) allocated 125,000 infants, by area, to vaccination or not and thereafter all infants were offered the vaccine through the nationwide immunisation programme. We report HBV serology from samples of GHIS vaccinees and unvaccinated controls, and from children born later.

**Methods:**

During 2007–08, 2670 young adults born during the GHIS (1986-90) were recruited from 80 randomly selected villages and four townships. Only 28% (753/2670) could be definitively linked to their infant HBV vaccination records (255 fully vaccinated, 23 partially vaccinated [1–2 doses], 475 not vaccinated). All were tested for current HBV infection (HBV surface antigen [HBsAg]) and, if HBsAg-negative, evidence of past infection (HBV core-protein antibody [anti-HBc]). HBsAg-positive samples (each with two age- and sex-matched HBsAg-negative samples) underwent liver function tests. In addition, 4613 children born since nationwide vaccination (in 1990-2007) were tested for HBsAg. Statistical analyses ignore clustering.

**Results:**

Comparing fully vaccinated vs unvaccinated GHIS participants, current HBV infection was 0.8% (2/255) vs 12.4% (59/475), p < 0.0001, suggesting 94% (95% CI 77-99%) vaccine efficacy. Among unvaccinated individuals, the prevalence was higher in males (p = 0.015) and in rural areas (p = 0.009), but adjustment for this did not affect estimated vaccine efficacy. Comparing fully vaccinated vs unvaccinated participants, anti-HBc was 27.4% (70/255) vs 56.0% (267/475), p < 0.00001. Chronic active hepatitis was not common: the proportion of HBsAg-positive subjects with abnormal liver function tests (ALT > 2 ULN) was 4.1%, compared with 0.2% in those HBsAg-negative. The prevalence of antibodies to hepatitis C virus was low (0.5%, 13/2592). In children born after the end of GHIS, HBsAg prevalence has remained low; 1.4% (15/1103) in those born between 1990–97, and 0.3% (9/35150) in those born between 1998–2007.

**Conclusions:**

Infant HBV vaccination achieves substantial protection against chronic carriage in early adulthood, even though approximately a quarter of vaccinated young adults have been infected. This protection persists past the potential onset of sexual activity, reinforcing previous GHIS findings of protection during childhood and suggesting no need for a booster dose. Nationwide infant HBV vaccination is controlling chronic infection remarkably effectively.

## Background

Chronic infection with hepatitis B virus (HBV) is a major cause of death from cirrhosis and liver cancer, chiefly in South-East Asia and sub-Saharan Africa [1,2]. Approximately 60 million people in Africa are chronically infected with HBV, mostly acquired perinatally or in early childhood [3,4]. A safe, effective vaccine consisting of the HBV surface antigen (HBsAg) was introduced in the 1980s, although coverage was initially limited by cost [5]. Universal infant vaccination against HBV is now recommended, and over the past decade three-dose coverage of infants has increased greatly across low and middle income countries. It now exceeds 70% worldwide, and 90% in The Gambia [6]. Protection by infant vaccination against infection in early childhood should suffice to prevent almost all cases of chronic infection in adults and hence of cirrhosis and liver cancer from HBV. For, although immunity can wane, HBV infection that is first acquired after childhood is unlikely to become chronic [1]. However, the expected impact of infant immunisation needs to be monitored.

During 1986–90 the nationwide Gambia Hepatitis Intervention Study (GHIS) allocated 124,577 infants, randomised by area of birth, to HBV vaccination (58,803) or not [7,8]. Since the end of recruitment into GHIS in February 1990 there has been universal nationwide infant HBV vaccination as part of the Expanded Programme on Immunisation (EPI). Prior to the GHIS, a study of infant vaccination against HBV in the two Gambian villages of Keneba & Manduar had begun in 1984, using historical controls. Both studies and the stepped-wedge design and sample-size calculation for the GHIS have been described previously [9-11]. Periodic follow up of vaccinees in both studies has found evidence of waning anti-HBs antibody levels and of substantial numbers having had an HBV infection (as indicated by persisting antibody to the HBV core protein), generally without any persistent clinical hepatitis. Despite these breakthrough infections, however, high vaccine efficacy against chronic HBV infection continued throughout childhood [12-21].

Although several studies have established vaccine efficacy into adolescence, most are in Asia where the predominant HBV serotypes differ from those in Africa and some 40% of chronic infection is acquired perinatally from the mother, whereas in Africa child-to-child transmission predominates [22-24]. It has not been established whether protection by infant vaccination continues into adult life, when repeated sexual exposure is likely.

To assess the long-term efficacy of infant HBV vaccination, in 2007-08 we tested sera of a number of subjects in the GHIS cohort (born 1986–90), to compare HBV markers in fully vaccinated individuals versus unvaccinated concurrent controls. In addition, to assess the effectiveness of the subsequent Gambian nationwide immunization program, we surveyed a sample of children born since 1990, irrespective of their vaccination status.

The serum samples from those born during 1986–90 were tested not only for markers of past and present HBV infection but also for infection with the hepatitis C virus (HCV), another major infective cause of cirrhosis and liver cancer in this population [25]. Among Gambians born around the middle of the 20^th^ century about 3% became chronically infected with HCV, possibly from non-sterile injections, and approximately 20% of liver cancers in later middle age are HCV-related [26-28]. In younger generations, however, iatrogenic exposures should have been reduced by sterile precautions, so the prevalence of chronic HCV infection should be lower.

## Methods

**Figure 1:**
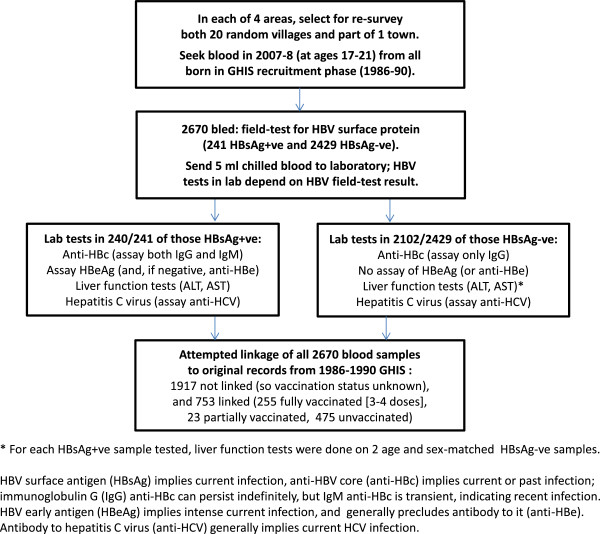
Flow diagram of the study.

This study is based on two cross-sectional surveys in 2007–08. One, described in Figure 1, was of vaccinated and unvaccinated young adults born during 1986–90 and therefore part of the GHIS, to assess the long-term efficacy of infant vaccination against current HBV infection and chronic active hepatitis (CAH, as indicated by alanine transaminase [ALT] elevation to greater than the upper limit of normal [ULN: 2 standard deviations above the population log mean]). We also tested these same individuals for anti-HBV-core-antibody (anti-HBc), which indicates past or current infection with the hepatitis B virus, and for anti-HCV antibody, which indicates current infection with the hepatitis C virus.

The other survey was of children born since 1990, and hence potentially covered by the national HBV vaccination program (which has been included in the nationwide Gambian Extended Program on Immunisation [EPI] since 1990), to assess the effectiveness of the national program against current HBV infection.

During 1986–90, The Gambia was divided into 40 areas, and vaccination began at different times in different areas. For the present study we selected four of these areas (two in Central River region, two in Upper River region) non-randomly, so that two (one from each region) were early adopters of HBV vaccination, starting in 1987, and two were late adopters, starting in 1989. In each area we then randomly selected for the present surveys 20 villages along with parts of the main town. The present survey of children born during 1986–90 took place in all four areas, but the survey of children born since 1990 was restricted to the 40 villages in Central River region.

Recruitment took place through mobile clinic days. We held community consent meetings prior to recruitment at each site. Participants passed through three desks at the mobile clinic: one for registration, one for blood collection and HBsAg field-testing, and one (at which non-study clinical services were also available) for communication of the test results.

All local residents aged 1–22 years were invited to participate. Written informed consent was obtained from participants or, where participants were children, a parent or guardian. Local village assistants contacted households in advance and on the day to identify participants. If available, census data could be used to help identify potential participants. We collected a 5ml blood sample from all born during 1986–90, and hence in the GHIS. We took only a finger-prick sample for HBsAg field-testing from those born since 1990, then sought a 5ml blood sample from those who field-tested HBsAg-positive, and from the mother of any person (born in 1986–90 or since 1990) found to be HBsAg positive. Each 5 ml blood sample was placed immediately into a cool box, and transported to the laboratory at the end of each day for processing that evening.

Figure 1 describes the survey of those born during 1986–90. Among them, the primary virology measurements were HBsAg (indicating current HBV antigenaemia), anti-HBc (indicating past or current HBV infection), and anti-HCV (indicating current hepatitis C infection). HBsAg was tested in the field by immunochromatography (Abbot Determine), a highly sensitive and specific test, and was not re-tested. All other serology was done at the main MRC laboratory in Fajara, where anti-HBc was measured by ELISA (DiaSorin, Sallugia). The main anti-HBc assay was of the long-lived IgG antibody (indicating past infection), but HBsAg-positive samples were also tested for the shorter-lived IgM antibody (indicating recent infection). HBsAg-positive samples were further tested for the early antigen (HBeAg), which indicates high infectivity, or, if this was absent, for antibody to it (anti-HBe). Anti-HCV was measured by immunoassay (AxSym analyser, Abbott).

To assess chronic active hepatitis (CAH), all HBsAg-positive samples (with two age-sex-matched HBsAg-negative samples) underwent liver function tests (ALT and AST) in Fajara (VITROS DT60-II analyser, Johnson & Johnson), with ALT > 2xULN used to define it.

Study forms were completed in the field, checked and transferred to the main MRC unit for double entry. Demographic information (age, sex, ethnicity and area of recruitment) was collected to allow linkage to original vaccination records in the GHIS database. Linkage was done, blind to vaccination and HBsAg status, by the program Reclink2 and by a separately written SQL program; consolidation of their findings was finalised before unblinding. Linkage was classed as reliable when all major information available was consistent with a single original record.

The study was designed to assess the continued efficacy of infant HBV vaccination. Odds ratios (ORs) and their 95% confidence intervals (CIs) were used to quantify relationships between vaccination status and serology. These were calculated by logistic regression, adjusted for study area, age, sex, and urban or rural location. Chi-squared tests were used to assess trends or heterogeneity. Statistical analyses used STATA 11.0 (StataCorp, Texas).

### Role of the funding sources

The sponsors had no role in the design, data collection, analysis, interpretation, writing or decision to publish this report. Ethical approval for the study was from the Gambian Government and The Gambia Government/MRC Ethics Committee (L2008.05, SCC1084v2).

## Results

### GHIS Recruits

2670 people born in 1986–90 (and hence possibly in the GHIS) were recruited, but reliable linkage to the original GHIS records of vaccination in infancy was possible for only 753 (28%). Recruitment was approximately balanced between the four study areas: Basse 905 (34%), Mansakonko 633 (24%), Farafenni 572 (21%), and Gambisara 560 (21%). Of all participants, 1751 (66%) were female and 1681 (63%) were recruited from villages rather than towns. Regardless of HBV vaccination history, the prevalence of current hepatitis C virus infection among these young adults, as indicated by anti-HCV antibody, was 0.5% (13/2598).

The prevalence of current hepatitis B infection, as indicated by HBsAg positivity, was 0.8% (2/255) in those fully vaccinated with 3 or 4 doses in infancy, 12.4% (59/475) in those unvaccinated, and 17.9% (4/23) among the few who had been only partially vaccinated (heterogeneity p < 0.00001) (Table 1). The crude odds ratio for HBsAg positivity comparing those fully vaccinated versus those unvaccinated was 0.06, suggesting 94% vaccine efficacy (with 95% CI 77-99%). Logistic regression was used to assess the impact of potential confounders on the association between vaccination status and HBsAg positivity, but adjustment for recruitment area, age, sex, and urban or rural location did not alter the odds ratio. Comparing fully vaccinated and unvaccinated subjects, the prevalence of past or present infection (as indicated by HBsAg positivity or IgG antibody to the HBV core protein) was 27% (70/255) vs 56% (267/475), p < 0.00001. Thus, although the prevalence of past or present infection among young adults is approximately halved by infant vaccination, many of the fully vaccinated participants had at some time been infected.

**Table 1:** HBsAg status in early adult life by number of doses of HBV vaccine in infancy among GHIS participants with reliable original vaccination records still available

	**HBsAg + (%)**	**95% CI**	**Odds ratio**
**HBV vaccine doses**			
0	59/475 (12.4%)	9.6 - 15.6	1
1 (partially vaccinated)	2/ 13 (15.4%)	1.9 - 45.4	1.28
2 (partially vaccinated)	2/ 10 (20.0%)	2.5 - 55.6	1.76
3 (fully vaccinated)	1/ 63 ( 1.6%)	0.4 - 8.5	0.11
4 (fully vaccinated)	1/192 ( 0.5%)	0.1 - 2.9	0.04
Total	65/753		-

p (trend) <0.0001.

Among those in either group (vaccinated or not) who were currently infected (HBsAg-positive), only 8% (5/65) had IgM antibody to the HBV core protein (indicating that the onset of infection was only recent). Of these 65 samples, 17 (26%) also tested positive for the early antigen (HBeAg-positive, indicating intense infection), 46 (71%) had antibody to the early antigen, and only 2 (3%) had neither the early antigen nor antibodies to it detected.

HBsAg positivity was strongly associated with ALT elevation, indicating some impairment of liver function (Table 2). Ignoring vaccination status and comparing all HBsAg-positive samples vs matched HBsAg-negative samples, the odds ratio for having ALT above the upper limit of normal (ULN) was 7.1 (26/218 vs 10/472, p < 0.0001), and the odds ratio for having chronic active hepatitis, as indicated by ALT > 2xULN, was 34.1 (9/218 vs 1/472). Although the numbers were small, chronic active hepatitis was, as expected, even more highly correlated with intense HBV infection, as indicated by HBeAg positivity.

**Table 2:** ALT elevation by HBsAg status in early adult life, comparing all HBsAg + cases vs a matched sample of twice as many HBsAg- controls 1986–90 births tested in 2007–08; analyses ignore vaccination status

	**HBsAg + cases (n = 218)**	**HBsAg- controls (n = 472)**	Odds ratio*
ALT category*			
Below control mean	76 (34.9%)	288 (61.0%)	1
Mean to ULN	115 (52.8%)	173 (36.7%)	2.5
ALT 1-2x ULN	18 ( 8.3%)	10 ( 2.1%)	6.8
ALT >2x ULN	9 ( 4.1%)	1 ( 0.2%)	34.1

p (trend) <0.0001.

*In HBsAg- controls, mean ALT is 16 IU/L and upper limit of normal (ULN) is 35 IU/L.

### Participants born since 1990

Among those born since 1990 (since the beginning of the nationwide vaccination program, which reaches most but not all of the population) the overall prevalence of current HBV infection, as indicated by HBsAg positivity, was only 0.5% (24/4613), about as low as among the fully vaccinated controls born in 1986–90. When stratified by 5-year age group there was an increase from 0.2% in those <5 years of age to 1.8% in those >15 years of age. (Table 3) Those born since 1997 were 1–9 (median 5) years of age when we surveyed them, and among them 9/3506 (0.3%) were HBsAg-positive.

**Table 3:** HBsAg positivity by age when surveyed in 2007–08 (and hence by year of birth) among 4613 children born since nationwide vaccination began in 1990 in The Gambia

**Years of age in 2007–08 (and median birth year)**	**HBsAg positivity/number tested (%)**
1- 5 (2005)	3/1921 (0.2%)
5- 9 (2001)	6/1591 (0.4%)
10-14 (1996)	10/ 825 (1.2%)
15-17 (1993)	5/ 276 (1.8%)
**1-17 (2002)**	**24/4613 (0.5%)**

p (trend) <0.0001.

There were only five HBsAg-positive children born since 1990 for whom we could find records showing they had definitely been vaccinated in infancy against HBV, and these were accounted for by only two mothers (three siblings in one family with an HBsAg positive mother and two in another family with an untested mother). There were no other associations with HBsAg positivity apart from the decrease with year of birth.

**Figure 2:**
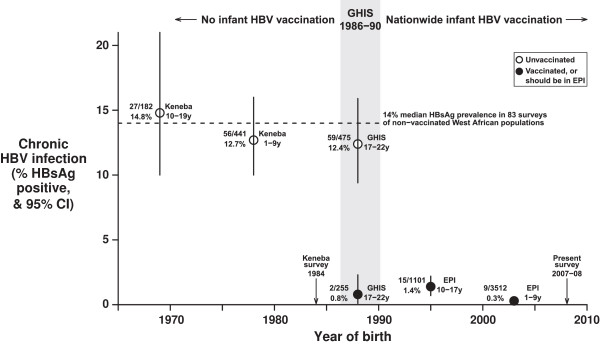
**Prevalence of chronic HBV infection in The Gambia, by year of birth: born before routine infant HBV vaccination; vaccinees and controls born during the Gambia Hepatitis Intervention Study (GHIS) in 1986–90; born since nationwide infant HBV vaccination. Key:** Number positive for HBV surface antigen (HBsAg)/number surveyed, % positive, and range of ages in years (y) when surveyed. Open circles = unvaccinated, closed circles = vaccinated in infancy (or supposed to be, in the Gambian EPI). **Keneba:** Unvaccinated historical controls (one village, children surveyed in 1984 at ages 1–19 years). **West Africa median:** Median of survey-specific HBsAg prevalences in 83 population surveys of unvaccinated children or adults conducted between 1990–2009 in West Africa (Merrill & Hunter, 2011). **GHIS:** Vaccinees and concurrent controls in the Gambia Hepatitis Intervention Study of children born in 1986–90, and surveyed for the present study in 2007–08, at ages 17–22 years, 0 · 8% vs 12 · 4% p < 0 · 00001. **EPI:** Population sample of children born since 1990 and surveyed for the present study in 2007–08 to assess the effectiveness of nationwide infant HBV vaccination (in the Gambian Expanded Programme on Immunisation), 1990–97 births 1 · 4% vs 1998–2007 births 0 · 3%, p < 0 · 00001.

The consistency of the estimates of the efficacy of full infant vaccination in the present survey, in previous surveys of GHIS participants and (with historical controls) in surveys of the Gambian village of Keneba is shown in Table 4. A review of sero-prevalence rates in unvaccinated populations shows that the pre-vaccination HBV prevalence rates in Keneba and in unvaccinated GHIS controls are reasonably representative of those in many other parts of West Africa [29,30]: Figure 2. (In one other Gambian village, Manduar, the pre-vaccination HBV prevalence rate was somewhat higher.)

**Table 4:** Reports from The Gambia of infant HBV vaccination efficacy against chronic HBV infection (HBsAg+) at various ages in childhood

	**Number HBsAg+/number tested (%)**		
**Age (years)**	**Vaccinees**	**Controls**	**% vaccine efficacy (and 95% CI)**
Nationwide GHIS cohort, born 1986–90 and surveyed periodically in 1990–2008, vs concurrently born controls			
~4	4/720 (0.6)	78/816 ( 9.6)	94% (84–98)
~9	4/675 (0.6)	85/823 (10.3)	95% (84–98)
~15	2/492 (0.4)	51/420 (13.2)	97% (92–99)
~20*	2/255 (0.8)	59/475 (12.4)	94% (76–99)
**All ages**	**12/2142 (0.6)**	**273/2534 (10.8)**	**95% (mean)**
Keneba village, born 1984–2002 and surveyed in 2003, vs historical controls surveyed in 1984			
1-4	0/176 (0.0)	24/236 (10.2)	
5–9	2/203 (1.0)	32/205 (15.6)	
10–14	0/185 (0.0)	19/135 (14.1)	
15+	3/224 (1.3)	8/47 (17.0)	
**All ages**	**5/788 (0.6)**	**83/623 (13.3)**	94% mean**

*Present survey in 2007–08; 3 previous GHIS follow-up surveys took place during 1990–2004 [8,14,15]. Results for Keneba were from a survey in 2003 [13] of vaccinees born 1984–2002 and from a historical survey in 1984 of unvaccinated children.

**Age-specific results are unstable due to small numbers.

## Discussion

This study shows that full infant HBV vaccination does protect strongly against chronic HBV infection (as indicated by detectable circulating levels of the HBV antigen), but protects less strongly against ever having HBV infection (as indicated by antibodies to the HBV core protein). Although in the present survey of people born in 1986–90 the sample of adolescents who had been vaccinated in infancy is relatively small (n = 255), it provides no evidence that the potential onset of sexual activity has as yet resulted in an increase in chronic HBV infection. Hence, the high efficacy now seen against chronic HBV infection in early adult life should result in high efficacy against HBV-related cirrhosis and liver cancer in future decades, even without booster doses for teenagers.

The main limitation of our survey was that record linkage of participants to their original vaccination records was unexpectedly difficult, and it was not possible to match the majority of participants born in the late 1980s to the database of original vaccination records. However, recruitment into our survey was unlikely to have been materially affected by vaccination status or HBV serology (both of which would generally have been unknown). So, there is no good reason to expect that, given the vaccination status, subclinical differences in serological status would bias the findings. A minor limitation was that a small number of anti-HBc test results (13%) were unavailable from the laboratory, so their values had to be imputed for calculation of the estimate of vaccine efficacy against ever having been infected.

Partial (one or two dose) vaccination appeared to be ineffective, but this conclusion is based on only 23 individuals (of whom four became HBsAg-positive), so it is not statistically reliable. Previous surveys of the GHIS cohort suggested substantial vaccine efficacy among those who received two doses of vaccine, but they too were based on relatively small numbers [12].

At early 1980s rates of chronic HBV infection, about 10% of unvaccinated five-year-old children would have been HBsAg-positive. The Gambian nationwide vaccination programme that began in 1990 may have taken a few years to achieve good coverage, but since 1997 HBV three-dose vaccination coverage has been about 90% nationwide. Hence, about 350 of the 3506 children in our sample of the whole population born since 1997 are likely to have been unvaccinated in infancy, or only partially vaccinated. If the 1980s infection rates among unvaccinated children had persisted, about 35 of these 350 would have been expected to be HBsAg-positive, whereas in fact only nine of these 3506 children were HBsAg-positive. This low prevalence suggests not only high efficacy among those vaccinated but also a significant degree of herd immunity.

Of the 24 HBsAg-positive children born since 1990, when nationwide HBV vaccination by the EPI started, some were probably infected perinatally from the mother. The evidence for this is two-fold: first, the mothers of some of these 24 HBsAg-positive children could be tested, and were more likely to be HBsAg-positive themselves (5/10) than would be expected from historical rates of adult prevalence; second, within these 24 children there were two clusters of infected siblings.

## Conclusions

### Chronic hepatitis B infection

The nationwide vaccination program that began in 1990 has been more effective than might have been predicted from extrapolation of the GHIS results. The program offers a birth dose of HBV vaccine plus a three-dose regimen of HBV-DPT vaccine, and has resulted in a massive decrease in the prevalence of chronic HBV infection between those born in the early 1980s and those born in the present century. Transmission from mothers may well account for the majority of the few remaining chronic infections in infants. As most chronic infection is acquired in early childhood, the significant downward trend in prevalence by year of birth since 1990 implies increasing effectiveness in the years since adoption in 1990 of universal HBV vaccination in The Gambia. This is partly because Gambian EPI coverage rates are better now than in the early 1990s, and partly because of herd immunity among children. High levels of infant vaccination will, however, continue to be needed, as for many decades there will still be significant numbers of people chronically infected with HBV in The Gambia and its neighbours.

### Chronic hepatitis C infection

The major decrease in chronic hepatitis B virus infection because of infant vaccination has been accompanied by a substantial decrease in chronic hepatitis C virus infection, probably due to improved sterile precautions in medical use of needles. Among the controls in a case–control study of adults in The Gambia there was a substantial prevalence of HCV (around 5%, 10/190) among those born before the 1950s but a much lower prevalence (0.5%, 1/192) among those born later [28]. In the present survey of young adults born during 1986–1990 the prevalence of HCV was again only 0.5% (13/2598), confirming the low HCV prevalence in recent generations. The present study tested much larger numbers of Gambians than any previous study and involved a representative community sample. Comparison of the results among older adults in other studies with those among young adults in the present study suggests a birth cohort effect involving almost a ten-fold decrease in the prevalence of HCV in The Gambia. This suggests that, in The Gambia as in many other countries, the mid-century epidemic of iatrogenic HCV infection from unsterilized needles has been controlled by the sterile procedures of the last quarter of the century. So, although HBV and HCV are causes of almost all liver cancer and cirrhosis deaths today in The Gambia, both should largely vanish over the coming decades.

## Competing interests

The authors declare that they have no competing interests.

## Authors’ contributions

The study was designed and conducted by AH, MM, TP, & HW. TP & EW collected and managed the study data. AH, TP, & EW analysed the study. AH, YL, MM, TP, EW, & HW wrote the paper. All authors reviewed the report and agree with its contents. TP had full access to all the data in the study and responsibility for the final decision to publish. All authors read and approved the final manuscript.

## Pre-publication history

The pre-publication history for this paper can be accessed here:

http://www.biomedcentral.com/1471-2334/14/7/prepub
